# Review of Artificial Intelligence Applications for Virtual Sensing of Underground Utilities

**DOI:** 10.3390/s23094367

**Published:** 2023-04-28

**Authors:** Kunle S. Oguntoye, Simon Laflamme, Roy Sturgill, David J. Eisenmann

**Affiliations:** 1 Department of Civil, Construction, and Environmental Engineering, Iowa State University, Ames, IA 50011, USA; oguntoye@iastate.edu (K.S.O.); sturgill@iastate.edu (R.S.); 2 Department of Agricultural and Biosystem Engineering, Iowa State University, Ames, IA 50011, USA; djeisen@iastate.edu

**Keywords:** artificial intelligence, virtual sensing, underground utilities, buried utilities, data fusion, knowledge database

## Abstract

Accurately identifying the location and depth of buried utility assets became a considerable challenge in the construction industry, for which accidental strikes can cause important economic losses and safety concerns. While the collection of as-built utility locations is becoming more accurate, there still exists an important need to be capable of accurately detecting buried utilities in order to eliminate risks associated with digging. Current practices typically involve the use of trained agents to survey and detect underground utilities at locations of interest, which is a costly and time-consuming process. With advances in artificial intelligence (AI), an opportunity arose in conducting virtual sensing of buried utilities by combining robotics (e.g., drones), knowledge, and logic. This paper reviewed methods that are based on AI in mapping underground infrastructure. In particular, the use of AI in aerial and terrestrial mapping of utility assets was reviewed, followed by a summary of AI techniques used in fusing multi-source data in creating underground infrastructure maps. Key observations from the consolidated literature were that (1) when leveraging computer vision methods, automatic mapping techniques vastly focus on manholes localized from aerial imagery; (2) when applied to non-intrusive sensing, AI methods vastly focus on empowering ground-penetrating radar (GPR)-produced data; and (3) data fusion techniques to produce utility maps should be extended to any utility assets/types. Based on these observations, a universal utility mapping model was proposed, one that could enable mapping of underground utilities using limited information available in the form of different sources of data and knowledge.

## 1. Introduction

Accidental utility strikes cost the United States approximately 30 billion dollars in damages in 2020 alone. These damages arise from direct (e.g., facility repairs), indirect (e.g., business closure, medical expenses), and societal (e.g., environmental pollution and traffic delays) costs. To promote safe excavation practices, the US utilizes the One-Call system [1], where excavators express intentions to dig and wait for concerned utility owners/operators to mark out the location of their facilities and indicate safe excavation areas. Yet, a recent study on the performance of the One-Call system in North Carolina highlighted challenges in the operation of the One-Call system to include prolonged wait times by operators, inaccurate locates, and low sensitization on excavation procedures among excavators [2]. The general inaccuracy and/or inefficiency of underground utility location practices is an important root cause of these accidental utility strikes [3].

A good share of investigation and locating practices employed are based on evidence of utilities, here termed evidence-based mapping, and often requires making inferences about the location of buried utility assets from the position of aboveground appurtenances. Evidence-based mapping also includes the use of any available information, such as as-planned and as-built maps. Nevertheless, while it is known that as-planned maps are often highly inaccurate, as-built maps may also be unreliable [4]. In particular, a recent survey reported that more than half of the utilities struck were missing from the available utility maps [5]. Another set of locating practices involves the use of nondestructive evaluation (NDE) techniques such as ground-penetrating radar (GPR) and acoustic emission (AE). However, these techniques can be costly and have equipment-specific challenges that limit mapping accuracy [6]. In particular, GPR is versatile in detecting metallic and non-metallic utilities, but performs poorly over highly conductive soils and the output data are complex to analyze; AE-based techniques performs well for non-metallic utilities, but is highly sensitive to noise [7].

Subsurface utility engineering (SUE) incorporates a classification approach for utility data quality levels to serve as a decision-support tool in the construction industry, ranging from quality-level A (high quality, low uncertainty) to quality-level D (lower quality, high uncertainty) [8]. Quality-level A data usually require sophisticated and more expensive mapping techniques [9]. Surveying visible (e.g., ground-level) appurtenances, akin to evidence-based mapping, is considered quality-level C data. On the other hand, mapping techniques that leverage NDE techniques typically output quality-level B data. These mapping practices are still labor intensive [10], thus contributing to prolonged mapping time, expenses, and inevitable human error, causing inconsistent data reliability.

A solution to improve mapping speed and accuracy is the automation of the mapping process through virtual sensing, where the location of utilities is determined by strategically combining limited information, including known utility positions and multi-source data. In the virtual sensing of underground utilities, the use of artificial intelligence (AI) tools becomes critical in creating inferences from available information in order to develop projected utility maps. The overarching goal of this paper is to review recent advances in the use of AI in automating the underground utility mapping process and recommend a pathway to implementing virtual sensing in future underground utility investigation approaches. Therefore, the main scientific contribution lies in the proposed universal underground utility mapping model developed to address the identified critical gaps in the consolidated literature.

The review of literature for this manuscript was conducted systematically by searching for all articles studying AI-aided mapping of underground utilities. The findings from this literature review were categorized within the areas of aerial mapping, terrestrial mapping, and data fusion of multiple data sources. Literature was also collected to support the proposed approach of using virtual sensing to investigate underground utilities. The categorized literature was then reviewed in deeper detail to prepare the narrative provided within this paper and the proposed method described herein.

**Figure 1:**
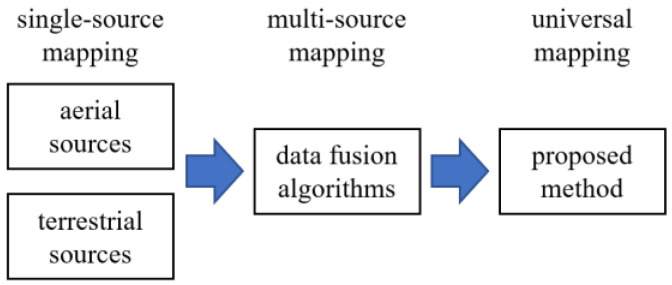
Organization of the paper.

This paper begins with a review of AI applications in underground utility mapping using single-source observations. As such, the next two sections review applications of aerial mapping, which includes the use of drones and satellite imagery, and applications of terrestrial mapping, which mostly concerns the use of NDE techniques. For clarity, the use of AI with photographic evidence is lumped in the aerial-based review. After, AI techniques leveraging multi-sourced data to create utility maps are reviewed. Finally, a path forward to conduct accurate virtual sensing of buried utilities is proposed, here termed universal utility mapping. The organization of the paper is illustrated in Figure 1.

## 2. Aerial Mapping

Aerial mapping (AM) of mapping underground utility typically consists of locating aboveground utility appurtenances to infer the likelihood of buried utility based on the utility flow of operations and industry rules. AM techniques are known to be cost-effective and non-intrusive [11]. AI in applications to AM is typically used as either (1) a computer vision tool to identify the assets or (2) a utility-mapping tool based on inference or logic rules to identify assets and predict the underground network. While it was argued by White et al. [12] that the concept of AM is more suitable for fluid-carrying utilities that depend on a gravity-flow rather than being pressurized, other works demonstrated the potential of AM to be successful for both fluid and non-fluid carrying utilities. For instance, Hassan et al. [13] generated an electrical utility map based on the location of transformers and poles using geographic information system (GIS) data.

One of the limitations of generating a utility map with the AM technique is its reliance on a knowledge database. While standards exist to harness such databases, such as those from the National Electric Safety Code and American Water Works Association, various uncertainties could lead to high variability in the accuracy of an automated process, including non-compliance [14] among utility operators and the presence of abandoned or out-of-service aboveground assets [15]. Additionally, the use of AM as an automated process was widely researched and demonstrated with manhole assets, but applications with other utility classes are still lacking.

**Figure 2:**
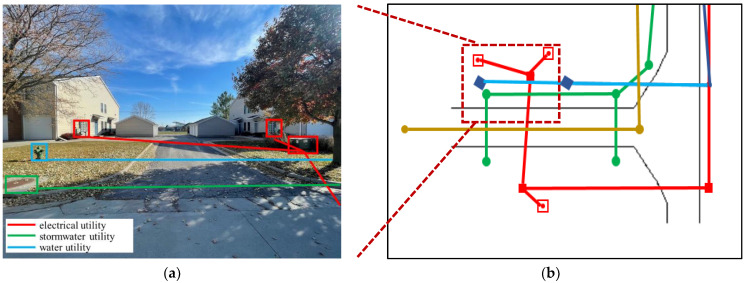
(**a**) Street-level image showing positions of utility appurtenance in rectangular boxes and the underground utility asset location in continuous lines; (**b**) utility map at the given location.

A popular method to automate AM mapping of aboveground assets is through computer vision algorithms. This task consists of identifying key utility assets from pictures. Figure 2 illustrates an example through a street-level image, showing identifiable utility-related appurtenances and the position of underground utilities at that location.

Potential sources of image inputs for AM include satellite imagery, aerial photographs, mobile images, photogrammetry, and light detection and ranging (LiDAR) technology collection. In particular, the use of satellite and aerial imagery can yield improved object recognition performance due to their improvement in image resolution [16]. As an example, Shermeyer et al. [17] found that the improvement in satellite imagery resolution from 30 cm/pixel to 15 cm/pixel upgraded the mean average precision of their object detection algorithm from 13% to 36%. Related to the application of satellite and aerial imagery to mapping utilities, Mnih et al. [18] applied a neural network approach to learn and detect road patterns. The detection of road patterns can be critical in mapping utilities as these sets of infrastructure are typically co-located [19]. There are additional examples of utility mapping using imagery. Bartoli et al. [20] reconstructed urban drainage networks by detecting manhole covers using a circular detection filter on orthophotos and satellite images. Pasquet et al. [21] identified the presence of underground utilities by locating manhole covers and stormwater drains from high-resolution aerial images using a linear support vector machine (SVM) classifier and a histogram of oriented gradients (HOG) that performed similarly to the circular detection filter used in Bartoli et al. [20].

Other works investigated asset detection using cameras. In particular, Timofte et al. [22] used single and multi-view computer vision techniques combined with GPS data to detect manhole covers from pictures obtained from cameras mounted on vans. Santos et al. [23] identified storm drains and manholes from street-level color RGB images by integrating the deep-learning computer vision model. Moy de Vitry et al. [24] proposed a multi-view detection method to improve sewer-inlet location detection rate in images obtained from stitched and undistorted unmanned aerial vehicle (UAV) images. Al-Kaff et al. [25] reviewed literature incorporating computer vision algorithms in UAVs for autonomous inspection, surveillance, and mapping, and Liu et al. [26] proposed a deep convolution neural network for detecting small manhole covers also captured by UAV.

Utility asset detection from point cloud data was also conducted, for instance, through the use of LiDAR. For example, Yu et al. [27] proposed a framework that automatically detects manhole covers from mobile laser scanning data; Turkan et al. [28] proposed the use of a wavelet neural network to compress point cloud data and store asset inspection features; Wei et al. [29] experimented with improved HOG symmetry features combined with SVM to detect and identify manhole covers; Gouda et al. [30] developed a fully automated process for identifying electric light poles from LiDAR point cloud data.

Other works explored automatic mapping following asset identification. Early works by Osman et al. [31] established a semi-automated system for decision-making in routing new utilities to avoid clashes with other existing utilities, constraints from surrounding elements, and other physical constraints. They proposed a three-branch framework; ontology knowledge formalization, communication and knowledge sharing with GIS, and reasoners using AI-fuzzy logic to assist in making an informed decision in selecting the best utility route. Li et al. [14] proposed a framework to automate utility location verificaton checks, a crucial step in minimizing incidents such as the pipeline explosions. Textual data are extracted and formulated into a hierarchical order using the specification language model and a natural language processing (NLP) model. Spatial reasoning outputs computer-readable logic that automatically creates a GIS model used in evaluating the compliance of as-built utilities with regulatory rules [14]. Later works by Xu et al. [32] and Xu et al. [33] showed advances in using ontology and spatial reasoning in executing automated utility compliance checks. Chahinian et al. [34] mapped wastewater networks from manhole locations combined with logic rules. The utility map was constructed from the minimization of a cost function defined by the logic rules that included variables such as manhole edge proximity, the slope between manholes, and interactions between roads and buildings.

Overall, the use of AI in conjunction with AM processes includes a vast array of methods to conduct both asset identification and buried utility location. Yet, from the review, the vast majority of works focused on the detection of manholes and their use in utility mapping. This disproportionate attention precluded the applications of AM and AI techniques to other utilities, such as electricity, natural gas, and telecommunication. There is also a lack of frameworks that combine both the tasks of asset identification and utility location. To guide work in asset location and mapping, Table 1 lists several assets associated with utility types and their typical locations [35].

It is important to note from Table 1 that some assets belong to more than one utility class. While there exist physical methods to reduce uncertainties, such as investigating the type of utility by opening manholes, as carried out in Dou et al. [4] and Chen and Cohn [36], or accessing other visual information, such as identification icons or inscriptions, automated routing algorithms could help understand and cope with these uncertainties. This can be carried out, for example, through fuzzy logic [37]. Other commonly shared assets include valve covers, pedestals, and control boxes.

## 3. Terrestrial Mapping

Terrestrial mapping (TM), not including photography, is typically conducted to achieve higher levels of mapping accuracy than those attainable via AM techniques. These can include the use of geophysical investigations or intrusive measures to achieve SUE quality-level A mapping accuracy through techniques such as vacuum excavation [38]. This method is widely used in today’s utility locating practices [39,40]. However, intrusive exposures for mapping can be costly as it can potentially disrupt critical activities such as construction and traffic [41], especially where utilities are within transportation right of way. The invasive technique also requires close attention to details during excavation to prevent damage to buried assets. A solution is to adopt non-intrusive sensing systems and geophysical tools such as ground-penetrating radar (GPR), metal detectors, vibro-acoustic systems, radio-frequency locators, and geoelectrical-resistive sensing methods [42]. These sensing methods output SUE quality-level B data [8], which has the potential to be further refined using data fusion techniques [43,44].

However, non-intrusive sensing requires highly trained operators to assess and evaluate information from scanned data [45]. Data processing can also be challenging, even for experts [46]. AI techniques provide a promising solution for assisting the non-intrusive process by automatically and rapidly evaluating sensing data to support actionable decisions. This section of the paper reviews various AI algorithms used in automating the utility detection process from non-intrusive data collection methods. It should be noted that a significant number of works focused on using AI to assist GPR systems due to their popularity over other sensing devices and the complexity in GPR data interpretation.

**Figure 3:**
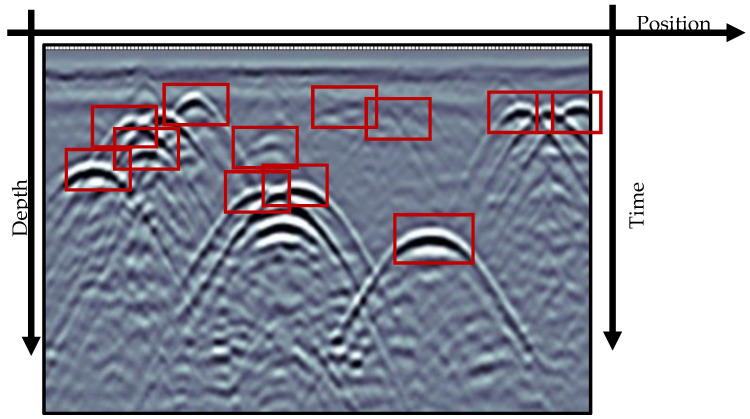
GPR radargram informing likely positions of utilities with hyperbolic signatures shown within red rectangles (adapted from Illingworth et al. [53]).

GPR-generated data can be complex to analyze. Figure 3 shows a typical generated scan ouptut with a gray background and different textures of hyperbolic signatures [47] bounded by red boxes. Typically, the hyperbolic signatures form by joining the discrete travel time of the electromagnetic waves as they pulse back from the targets [48]. Information such as the object’s depth and horizontal position is estimated using the hyperbolic signature’s spread, position in radargram, and degree of resolution. Yet, the manual reading of radargrams is often challenging due to the intrinsic differences in material properties of targets and soil characteristics. Several AI tools were developed and applied to assist the interpretation of GRP-collected data, in particular for applications regarding underground utilities [49,50,51,52].

One popular tool is the Hough transform (HT), used to extract geometrical features in images [53]. It was used by Capineri et al. [54] in locating the position of buried objects represented by hyperbola in GPR radargrams. The HT was also used in Al-Nuaimy et al. [55], where the HT was combined with a backpropagating neural network to detect buried utilities and other solid objects. Al-Nuaimy et al. [56] proposed an adaptive non-accumulated Hough transform (ANHT) to reduce the memory demand relative to the conventional HT and demonstrated that the technique could be applied to detect linear structures such as pipes and cables. Modified HT variants were proposed. For instance, Simi et al. [57] developed the randomized HT that yielded underground utility detection accuracy greater than 85% over different ground conditions such as asphalt, grass, and concrete; Al-Nuaimy et al. [55] used a modified HT to detect targets in a GPR radargram with a significantly low signal-to-noise ratio; and Maas et al. [58] combined the Viola–Jones learning algorithm with the HT to separate regions of interest and extract vertices and locations of the hyperbola. Other works adopted the HT technique for recognizing, localizing, and evaluating hyperbolic indications, wave velocity, buried pipe diameters, and asset depths in radargram [59,60,61,62].

It was argued in Chen and Cohn [62] that some early methods enabling automatic detection in GPR hyperbolic signatures were not suitable for on-site applications due to their computation complexity and unsatisfactory detection rate resulting from insufficient training. The authors proposed a probabilistic hyperbolae mixture model based on a classification expectation-maximization algorithm and a Bayesian information criterion for model selection. Compared to other HT, their proposed model completed the utility identification task in 1.7 s, while the HT completed a similar task in 226.1 s. In Dou et al. [63], a column-connect clustering algorithm was developed, which first discriminates targeted utilities represented as hyperbolas from the radargram background, with the algorithm yielding a 23% improvement in detection rate than for the pattern recognition technique used for a similar task in [58]. A variant of the column-connect clustering algorithm was investigated by Rosin, P. [64] that resulted in a 6% improvement in the model accuracy (F1-score).

Support vector machine (SVM) gained popularity in GPR data classification tasks. Zhang et al. [65] used SVM for the automatic extraction and classification of buried landmines from a GPR radargram; El-Mahallawy et al. [66] adopted an SVM classifier to identify underground utilities and showed 100% and 78% recognition accuracies in an ideal and noise-degraded environment, respectively; Lu et al. [67] proposed to combine a discrete wavelet transform, fractional Fourier transform, and SVM for classifying subsurface objects material from GPR data; Terrasse et al. [68] detected buried gas pipes using an SVM classifier and reported a 96% detection rate; Kaur et al. [69] combined HOG with SVM to map deteriorated reinforcement bars; Noreen et al. [70] automated buried pipe detections using HOG features clustered with an SVM algorithm; and Ozkaya et al. [71] fed features extracted from GPR radargram images using convolutional neural networks into an SVM algorithm to detect and classify hyperbolic signatures, material, shape and soil type.

Genetic programming (GP) was employed in automating GPR data processing. Pasolli et al. [72] proposed an automatic detection process that uses an iterative genetic algorithm to detect and locate the position of underground utilities in GPR radargrams. Kobashigawa et al. [73] adopted GP to detect buried unexploded ordnances in GPR radargrams. The GP algorithm performance was compared against different neural networks of varying hidden layers and showed improved performance over complex radargrams. Additionally, Harkat et al. [74] proposed to utilize a radial basis network with a multi-objective genetic algorithm to improve region classifications.

There is also a vast amount of research that focused on the use of neural networks in processing GPR radargrams. For example, Birkenfeld, S. [75] presented a non-fully connected neural network model that identifies interfering or incomplete hyperbolas. Lei et al. [46] proposed the faster region-based convolution neural network (R-CNN) and data augmentation to detect regions of buried objects in GPR images. Liu et al. [76] proposed a deep learning model, termed a single shot multi-box detector, to detect hyperbolae. When compared with the faster R-CNN model adopted by Lei et al. [46], the technique showed a higher precision rate with lower computational time. Yamaguchi et al. [77] conducted the 3-D location of buried pipes in GPR radargrams using 3D-CNN. A 10% improvement in accuracy level was observed when compared to an SVM. The extracted positions, inclinations, and arrangement of pipes were visualized in a 3-D map using Kirchhoff migration. Liu et al. [78] identified limitations in using hyperbolic signatures as the primary representative feature for buried utilities. The 3D GPR scan images formed by stitching parallel scan data were used as input to a 3D CNN. The proposed method produced a detection accuracy of 82.7%, compared with 69% for CNN with 2D GPR input data and 61% to 73.5% for other methods that included the AlexNet, ResNet50, VGG16 and 19, and Triplanar algorithms.

While more limited, other works focused on non-intrusive techniques other than GPRs to detect buried utilities, such as metal detectors [79], vibro-acoustic sensing techniques [80,81], geoelectrical resistivity, and radio-frequency locators. For instance, Dutta et al. [81] automatically located the presence of underground utilities by using a novel pattern recognition and edge detection algorithm based on Canny filtering to analyze and identify hidden patterns from contour plots and sub-surface cross-sectional images in vibro-acoustic sensing data; Safatly et al. [79] reduced false-alarm rates during manual landmine investigation with metal detectors by developing a machine-learning model trained to discriminate between sounds produced when a landmine is present and not; and Vanijjirattikhan et al. [80] proposed a classification model trained on leakage sounds to improve leakage detection with acoustic techniques.

In summary, most of the reviewed literature in this section focused more on GPR because it remains the most used electromagnetic sensor for utility mapping. Other sensors demand AI applications to further improve data interpretation, such as vibro-acoustic and metal detectors that are used for non-metallic utility detection and rapid utility probing, respectively.

## 4. Data/Information Fusion

The previous two sections focused on the use of AI to interpret single-source data. In creating virtual sensors capable of mapping underground utilities based on limited information, one must consider the integration of multi-source data in order to improve the quality of the harnessed information and, thus, the accuracy of maps through a process known as data fusion [82,83,84,85]. For example, Dutta et al. [81] proposed to fuse GPR and vibro-acoustic data to combine sensing performance in reading through non-saturated soils (GPR) and saturated soils (vibro-acoustic), and Metje et al. [11] studied the potential of combining GPR, low-frequency quasi-static electromagnetic fields, and acoustic technologies to improve underground utility mapping accuracy. Generally, while the fusion of data is conducted through user-defined rules and metrics, the iterative process is automated for robustness over extensive knowledge and data space [86]. This section surveys different AI techniques used in fusing data in order to produce maps of underground utilities.

Fuzzy logic was used by Lanka et al. [37] as an approximate reasoning method to fuse data from different sources. The proposed model determines the probability of a utility to be located at any Cartesian point based on (1) proximity to manholes; (2) perpendicular distance from the point of interest to the nearest known utility line; (3) expert knowledge on a possible underground utility route; and (4) GPR data analysis. Another technique studied by Chen and Cohn [36] is based on Bayesian data fusion (BDF) to automatically produce refined utility maps by fusing GPR data, surveyed manholes, assumptions on pipe linearity, and utility records. In this research, manholes are investigated to determine the depths and orientations of the underground pipes. This study adopted the joint compatibility branch and bound algorithm developed by Neira and Tardos [87] to establish an initial map by matching utilities discovered from the manhole survey and GPR scan. Prior knowledge used in the BDF is deduced by matching connections in the as-built maps and GPR-detected points. With the Gaussian distribution assumed, the posterior probability of the BDF is estimated from prior and likelihood probabilities. The posterior map is automatically refined through an iterative process guided by a validation threshold.

Recent work by Zhou et al. [88] proposed a probabilistic pipeline mapping model that fuses records, pipe information from manhole covers, and remote sensing technologies such as GPR and electro-magnetic. The model maximizes the likelihood of fitting a pipeline from the set of detected points. By integrating the Bayesian information criterion, the proposed model avoids overfitting when classifying and fitting detected points into the most probable pipeline. The authors compared the performance of their algorithm with that proposed in Chen and Cohn [36] and demonstrated fewer mapping errors.

**Figure 4:**
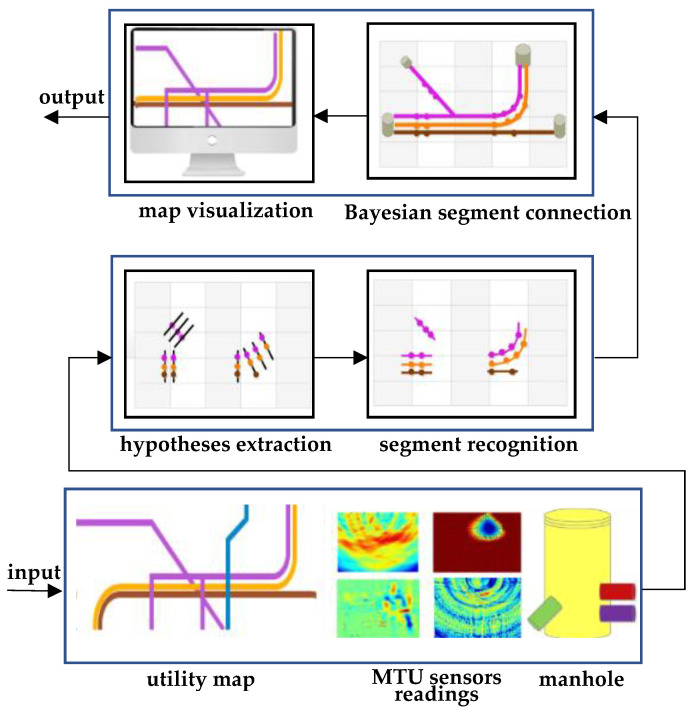
Data fusion workflow from Bilal et al. [89].

Additionally, Bilal et al. [89] mapped underground utilities with different probabilistic models. Figure 4 shows the sequence of workflow starting with maps, surveyed manholes, and sensor readings obtained from the mapping the underworld (MTU) initiative [11]. MTU is a United Kingdom initiative targeting the construction of highly accurate underground utility maps leveraging sensing data, such as GPR and vibroacoustic measurements, without excavation. The authors select candidate hypotheses from MTU sensor readings using a maximum likelihood probabilistic approach to recognize utility segments. Segment connections are completed using a Bayesian approach, and finally, the underground utility map is prepared for visualization.

Dou et al. [4] generated utility maps from the combination of information collected from manhole investigations and GPR data. The manhole investigation allows the utility to be characterized by their directional angle, depth, and material type. The probability of utilities routing along the observed directions is estimated with a Gaussian distribution. The extracted utility location hypotheses from GPR data reduces the degree of uncertainty of the different directions the utility can take beyond the observed range. The most probable connection between two utility ends (i.e., manholes) is derived by maximizing the product of different probabilities of utilities exiting both ends using integer linear programming (ILP). This model’s experimental result yielded a 100% accuracy (F1-score) on field data and an average of approximately 80% on synthetic data. The model’s dependency on investigating manholes as the starting or ending point renders it insufficient for broader applications.

Cai et al. [7,90] identified the limitations of the data-fusion techniques discussed in [36,81]. These limitations included overreliance on data sources, assumed distributions, lack of prior information, and difficulty in estimating prior probabilities. The authors further proposed a technique that fuses data from both sensor and non-sensor sources based on Dempster–Shafer’s theory. With GPR and as-built records as selected data sources, understudying the limitations and other uncertainties reveals the confidence level of every connection in each data source. Each connection is characterized using mass values assigned to the three keywords—existence, absence, and ignorance. The joint mass values from both data sources, thus, reveal every matched connection certainty and uncertainty level. While the models produced satisfying results when tested with an indoor experimental setup and simulated records, the assumption of pipe perpendicularity with the GPR survey pathway survey line is not always true for real-world data.

Dutta et al. [81] used dynamic Bayesian network to estimate the most probable 3D map of buried utilities using data from ground-excited vibroacoustic emissions, pipe-excited vibroacoustic emissions, and GPR systems. The authors adopted intelligent image-processing techniques to process individual sensor image data before fusing the extracted utility location and depth with the dynamic Bayesian network. The resulting model-generated map provided higher accuracy compared with utility records from Moravec, H.P. [83].

**Figure 5:**
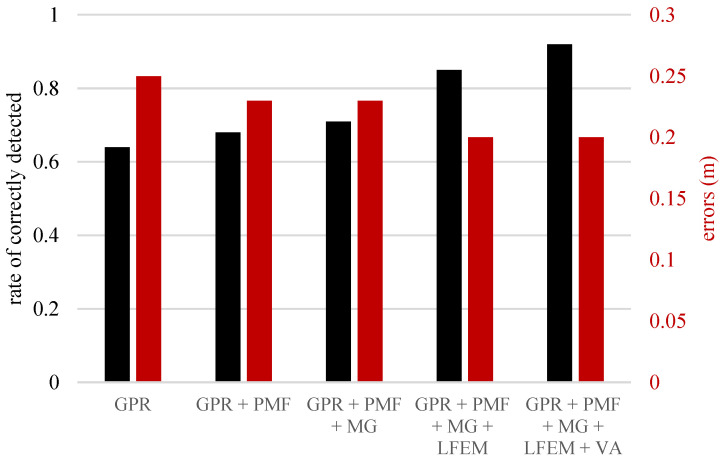
Graph of error and rate of correctly located utility. PMF– passive magnetic field, MG– magnetic gradiometer, LFEM– low-frequency electromagnetic field, VA– vibro-acoustics (adapted from Dou et al. [44]).

In Dou et al. [44], GPR, passive magnetic field, vibroacoustic, and magnetic gradiometer data were fused to improve a utility map’s accuracy. Data fusion was conducted using a novel marching cross-section algorithm and Kalman filter in marching scanned utility tracks. The bar plot in Figure 5 shows the progression and regression in detection rate and error, respectively, as the number of sensing modes increases.

The reviewed literatures combined different data sources, mostly GPR, utility records, manhole survey, and other sensors. One underlying common limitation to most work is the reliance on manhole surveys. Firstly, it restricts the applicability to manhole-related utilities, and secondly, it is burdensome to open up and determine the geospatial coordinates of every manhole in the area of interest at the same time. It should also be noted that techniques relying on as-built maps as prior knowledge can be challenging to apply when such information is unavailable or outdated.

## 5. Enabling Virtual Sensing of Underground Utilities

In the previous sections, we reviewed different applications of AI representing efforts to automatically map underground utilities, including those that employed sensing these utilities through the fusion of multi-source data. From the reviewed works, it is clear that the field made great progress at (1) detecting manhole locations and reconstructing utility maps using available knowledge from what manholes represent, whether it included physical investigation or not; (2) transforming complex GPR measurements into hypotheses on the location of utilities; and (3) fusing information obtained from manhole locations, GPR data, available as-built maps, and metadata such as those obtained through available records. Yet, it follows that important gaps exist in (1) mapping utilities from other assets, such as transformers and valves; (2) extending AI tools to extract useful information from other non-intrusive evaluation techniques; and (3) extending data fusion techniques to any utility assets/types (e.g., those listed in Table 1) and an array of different data sources.

**Figure 6:**
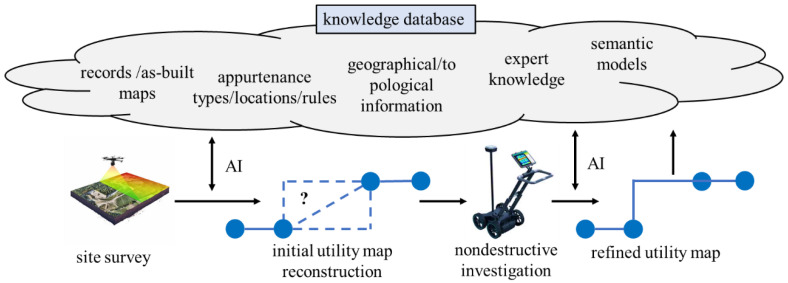
Proposed integrated model enabling virtual sensing of underground utilities.

Building on the surveyed literature, we see an opportunity in producing an integrated, universal utility mapping model that could enable mapping of underground utilities using limited information available in the form of different sources of data and knowledge. The proposed model is depicted in Figure 6. The process consists of, first, constructing an initial utility map based on a site survey. This initial map may include probabilistic information to improve the quality of initial guesses on utility locations. Afterwards, the map is refined as needed using non-intrusive evaluation techniques, such as GPR and vibroacoustic measurements. Importantly, a knowledge database would support the mapping process, which constitutes the central mechanism enabling virtual sensing. We foresee such a database to be open access and open sourced, with various stakeholders capable of modifying the inputs in order to improve the quality of the harnessed information and, thus, virtual sensing accuracy. The knowledge database is to include the following critical information.

**Records and as-built maps**: the readily available, recorded information on the location of utilities. These may include as-planned and as-built maps. Some of this information may be considered as “truth,” or at least assessed with a high probability of accuracy. As-built maps can be considered as a starting point in mapping utilities or used in guiding the non-intrusive process by comparing these maps with the initial reconstructed utility map [7,36].

**Asset types and rules**: a dictionary of possible asset types and asset-specific rules that can be used in mapping. These also include libraries of pictures that can be accessed for training computer vision algorithms. Rules associated with specific asset types, such as the depth and expected direction of utilities [35], will be used to create initial utility maps. These rules include fuzzy logic to determine the probability of utility routing.

**Geographical and topographical information:** a set of site-specific data relative to the geographical location and ground topography. This information includes local law pertaining to utility routing. Knowledge of the topography can be useful in guiding GPR inspections and establishing rules for gravitational systems (Chahinian et al. [34], for instance).

**Expert knowledge**: information input by field experts; for example, utility inspectors and managers. For example, the typical burial depth or possible deviations from assets, or known approximate placement and routing as a function of a given neighborhood based on prior investigations.

**Semantic models:** a set of models empowering the fusion of semantic data to improve the accuracy of the mapping process. These may include, for instance, the extraction and interpretation of utility burial rules/laws (Xu and Cai [32,33], for instance), and/or the direct interpretation of expert knowledge input.

The knowledge database cross-interacts both at the initial mapping level and non-intrusive mapping level through various AI rules, such as those reviewed in the previous sections. AI can be seen as a tool to extract useful information from the knowledge database and assist at creating utility maps. Its role spans from the computer vision algorithms used in detecting assets to data-fusion algorithms used in creating more accurate routing in the initial map. Once the refined map is created from the non-intrusive investigation campaign, new knowledge is created and fed back to the knowledge database. Ideally, this new information is used to update rules and/or available dictionaries of information.

## 6. Conclusions

This paper reviewed different techniques supporting virtual sensing of underground utilities leveraging advances in artificial intelligence (AI). In particular, the use of AI in aerial and terrestrial mapping of utility assets through visible appurtenances was reviewed, followed by a summary of AI techniques used in fusing multi-source data in creating underground infrastructure maps. Key findings from these sections identified the research gap in achieving accurate virtual sensing of utility assets. The biased attention of the majority of works to a single utility class or popular utility sensor, and limited data sources for the fusion process hindered the generalized application and high accuracy. Based on these findings, a universal utility mapping model was proposed. The model uses limited information available in the form of different sources of data and knowledge. It consists of first constructing an initial utility map based on a site survey, after which the map is refined as needed using non-intrusive evaluation techniques. Importantly, a knowledge database is used to support the mapping process, which constitutes the central mechanism enabling virtual sensing. Future work in the area could include the creation of an open-source knowledge database that could be populated, augmented, and altered by experts in order to refine the performance of AI systems at conducting virtual sensing of underground utilities.

## Figures and Tables

**Table 1:** Aboveground features of different utility classes (adapted from [35]).

Utility	Assets	Locations
Electricity	1, 2, 3, 4, 5, 6, 15, 17, 18	Sidewalk, property line, and toward buildings
Sewerage	3, 7, 9, 17, 18	Sidewalk, property line, and toward buildings
Stormwater	3, 6, 8, 17, 18	Along the road/right of way
Telecom.	1, 2, 3, 5, 6, 10, 11, 17, 18	Sidewalk, property line, along the road/right of way
Water	3, 12, 13, 16, 17,18	Sidewalk and toward buildings
Natural Gas	6, 13, 14, 17, 18	Sidewalk and toward buildings

1: Pedestal, 2: Control Box, 3: Manhole, 4: Transformer, 5: Electric Poles, 6: Trench Trail, 7: Cleanouts, 8: Storm Traps, 9: Culvert/Gate, 10: Antenna, 11: Closures, 12: Fire Hydrant, 13: Valve Cover, 14: Gas Meter, 15: Electric Meter, 16: Water meter, 17: Utility flags, 18: Field marks.

## Data Availability

Data sharing not applicable.
